# Mutation Analysis of the RPGR Gene in a Chinese Cohort

**DOI:** 10.3389/fgene.2022.850122

**Published:** 2022-03-31

**Authors:** Hong-Li Liu, Feng-Guan Gao, Dan-Dan Wang, Fang-Yuan Hu, Ping Xu, Qing Chang, Ge-Zhi Xu, Ji-Hong Wu

**Affiliations:** ^1^ Eye Institute, Eye and ENT Hospital, College of Medicine, Fudan University, Shanghai, China; ^2^ State Key Laboratory of Medical Neurobiology, Institutes of Brain Science and Collaborative Innovation Center for Brain Science, Shanghai Medical College, Fudan University, Shanghai, China; ^3^ Shanghai Key Laboratory of Visual Impairment and Restoration, Science and Technology Commission of Shanghai Municipality, Shanghai, China; ^4^ Key Laboratory of Myopia (Fudan University), Chinese Academy of Medical Sciences, National Health Commission, Shanghai, China

**Keywords:** retinitis pigmentosa, retinitis pigmentosa GTPase regulatory factor, gene mutation, genetic testing, genetic analysis

## Abstract

**Purpose:** The purpose of this study was to investigate the clinical and genetic characteristics of the retinitis pigmentosa GTPase regulatory factor gene (*RPGR*) in a Chinese cohort.

**Methods:** A retrospective analysis was performed on 80 subjects with *RPGR*-retinal dystrophy (*RPGR*-RD) for detailed genetic and clinical characterization. The panel-based next-generation sequencing of 792 causative genes involved in common genetic eye diseases was conducted in all individuals, followed by clinical variant interpretation. Information, including age, sex, geographic distribution, family history, consanguineous marriage, age at symptom onset, disease duration, best corrected visual acuity (BCVA), and complete ophthalmologic examination results, was collected.

**Results:** This cohort (41 men and 39 women) included 26 families (26 probands and their available family members) and 13 sporadic cases. The average age of these participants was 36.35 ± 17.68 years, and the majority of the families were from eastern China (28 families, 71.79%). The average duration of disease in the probands was 22.68 ± 15.80 years. In addition, the average BCVA values of the right and left eyes in the probands were 0.96 ± 0.77 and 1.00 ± 0.77, respectively. A total of 34 *RPGR* variants were identified, including 6 reported variants and 28 novel variants. Among these variants, NM_001034853.1: c.2899_2902delGAAG and c.2744_2745ins24 were considered *de novo* variants. The majority of the *RPGR* variants were classified as likely pathogenic, accounting for 70.59% of the variants (24 variants). The most common nucleotide and amino acid changes identified in this study were deletions (16 variants, 45.06%) and frameshifts (17 variants, 50.00%), respectively. Genetic analysis revealed that these *RPGR* variants were distributed in 10 different subregions of *RPGR,* and 70.59% of the *RPGR* variants (24 variants) were located in exon 15. Four *RPGR* variants, NM_001034853.1: c.2405_2406delAG, c.1345C > T, c.2218G > T and c.2236_2237delGA, occurred at a very high frequency of 28.21% (11 families) among 39 unrelated families.

**Conclusion:** This study expands the known mutational spectrum of *RPGR*, and we provide a new reference for the genetic diagnosis of *RPGR* variants.

## Introduction

The retinitis pigmentosa GTPase regulator gene (*RPGR*; OMIM: 312610), located on chromosome Xp11.4 with 25 exons, was first reported by Meindl et al. in 1996 (Meindl et al., 1996; Roepman et al., 1996a; Roepman et al., 1996b). This gene has a total length of 60 kb and encodes a protein with a series of six RCC1-like domains (RLDs). The encoded protein localizes to the outer segment of rod photoreceptors and interacts with retinitis pigmentosa GTPase regulator-interacting proteins (RPGRIPs) (Boylan and Wright, 2001; Hosch et al., 2011; Khanna et al., 2010; Khanna, 2018; Murga-Zamalloa et al., 2010). A total of 13 Gene Ontology terms (centrosome, ciliary basal body, Golgi apparatus, guanyl-nucleotide exchange factor activity, intracellular protein transport, intraciliary transport, photoreceptor outer segment, positive regulation of GTPase activity, protein binding, response to stimulus, sperm flagellum, and visual perception) are linked with *RPGR* according to data published in the Human Genome Mutation Database (HGMD, http://www.hgmd.cf.ac.uk/ac/index.php). Three major isoforms of human *RPGR* have been identified: *RPGR* ^ex1-19^, *RPGR* ^skip14/15^, and *RPGR* ^ORF15^ (Vervoort et al., 2000), which share a common N-terminal domain and RCC1-like domain, but their C-terminal domains are different (Kirschner et al., 1999).

A great deal of work has been conducted to uncover the essential effect of *RPGR* on the retina. According to published data (in January 2022) on HGMD, 303 mutations in *RPGR* can be associated with more than 20 eye diseases, including X-linked retinitis pigmentosa (XLRP, RP3) (Falls and Cotterman, 1948) and X-linked cone-rod dystrophy (Pinckers and Timmerman, 1981). Among these diseases, the most common presentation (>70%) associated with *RPGR* mutations is XLRP (Vervoort et al., 2000). The first clinical sign in most XLRP cases is night blindness, which may ultimately cause vision loss (Breuer et al., 2002). However, the clinical presentation is highly differentiated between affected men and women (Beltran et al., 2015). Specifically, the symptoms are particularly severe in affected men, in whom the disease is characterized by early onset and rapid progression of vision loss, resulting in legal blindness by the end of the third decade of life. Unlike men, most affected women show heterogeneous symptoms, ranging from asymptomatic electrophysiological abnormalities to very serious retinal diseases (Fishman, 1986), and legal blindness occurs at approximately 30–40 years of age (Bird, 1975).

Our previous study reveals that *RPGR* mutations, with a prevalence of 4% in the Chinese population, were ranked as the fourth most common genetic mutation in patients with inherited retinal dystrophy (IRD) (Gao et al., 2019a). This high prevalence of *RPGR*-retinal dystrophy (*RPGR*-RD) means that there is an urgent need for further research on its genetic characteristics. The present study aims to report novel genetic data on *RPGR* based on a Chinese cohort. The distribution and publication of this kind of data is helpful to molecular diagnostics professionals and ophthalmologists for interpreting the genetic testing results of patients affected by rare inherited retinal dystrophies. Moreover, gene therapy for RPGR-related retinal dystrophies is currently being tested in multiple studies, and the identification of patients who may benefit from this therapy is important.

## Methods

### Study Subjects and Ethics Statement

The study protocol was reviewed and approved by the Ethics Committee of the Eye and ENT Hospital of Fudan University. This study conformed to the tenets of the Declaration of Helsinki. A total of 80 subjects from 39 unrelated families with *RPGR*-RD, including 44 subjects from a previous article (Gao et al., 2019a), were recruited for this study. All individuals of Chinese descent were enrolled from the Eye and ENT Hospital of Fudan University between January 2017 and December 2021. Written informed consent was obtained from all participants or their guardians.

### Genetic Information

Genomic DNA was extracted from the peripheral blood of each affected subject and their available family members. The detailed genetic information of all subjects was obtained as previously described (Hu et al., 2019). A high-throughput target enrichment approach was designed to capture the exons and untranslated regions (UTRs) of 792 causative genes involved in common genetic eye disease (Supplementary Table S1). The panel was specifically constructed for this study by Beijing Genomics Institute (BGI, Inc., Shenzhen, China). Genomic DNA was sheared into different fragments containing coding exons, flanking intronic areas, and promoter regions, which were captured utilizing the Agilent SureSelect Target Enrichment Kit (Agilent Technologies, Inc., Santa Clara, United States). The enriched libraries were sequenced on the MGISEQ-2000 platform according to the manufacturer’s instructions. Sequencing reads were mapped against the reference human genome (hg38) utilizing a Burrows–Wheeler Aligner (BWA, http://bio-bwa.sourceforge.net/).

Four databases were employed for the further assessment of the identified variations: 1000 Genomes Project (http://browser.1000genomes.org/), dbSNP (http://www.ncbi.nlm.nih.gov/projects/SNP/), ESP6500 (http://evs.gs.washington.edu/EVS/), and ExAC (http://exac.broadinstitute.org). The potential deleteriousness of each variant with a minor allele frequency (MAF) < 0.1% was further confirmed. Moreover, three internet-based tools were utilized to predict potential pathogenic variations: the Sorting Intolerant from Tolerant (SIFT, http://sift.jcvi.org/), Polymorphism Phenotyping v2 (PolyPhen-2, http://genetics.bwh.harvard.edu/pph2/), and MutationTaster (http://www.mutationtaster.org/) programs. Then, the remaining potential deleterious variations were further screened to identify pathogenicity according to three databases: ClinVar (https://www.ncbi.nlm.nih.gov/clinvar/), HGMD (http://www.hgmd.cf.ac.uk/ac/index.php), and Online Mendelian Inheritance in Man (OMIM, http://www.omim.org/). The final set of variants were classified as pathogenic, likely pathogenic, variants of uncertain significance (VUS), benign, or likely benign in accordance with the American College of Medical Genetics (ACMG) and genomics guidelines.

### Clinical Information

Basic information of the subjects, including their age, sex, geographic distribution, family history, age at symptom onset, and disease duration, was collected for analysis. The complete ophthalmologic examinations performed on the probands included the following components: best corrected visual acuity (BCVA), slit-lamp biomicroscopy, wide-field fundus imaging (Optos PLC, Dunfermline, United Kingdom), fundus autofluorescence (FAF, Spectralis HRA COCT; Heidelberg, Germany), visual field assessment (VF, Humphrey Visual Field Analyzer, Carl Zeiss Inc., Dublin, CA, United States), swept-domain optical coherence tomography (Spectralis HRA + OCT, Heidelberg Engineering Inc., Heidelberg, Germany), and full-field electroretinography (ffERG) (Gao et al., 2019b; Hu et al., 2019).

All probands were clinically diagnosed by an experienced ophthalmologist according to their clinical symptoms and complete ophthalmologic examinations. The genetic diagnosis of individuals was further performed based on the clinical diagnosis and genetic variant interpretation.

### Classification

The probands were classified into group A or B according to their *RPGR* variant distribution. Probands with *RPGR* variants located in exon 15 were included in group A, and probands with *RPGR* variants located in the other regions were included in group B.

According to the BCVA (logarithm of the minimum angle of resolution, logMAR) of the eye with better vision, the vision of the probands was classified according to three levels. Vision impairment was defined as ≤0.5 logMAR; low vision was defined as 0.5–1.3 logMAR; and legal blindness was defined as >1.3 logMAR.

## Statistical Analysis

All analyses were performed with SPSS software (SPSS 22.0, SPSS Science, Chicago, IL, United States). A *p* value of <.05 was considered statistically significant. All data were first classified as measurement data or count data. The measurement data are presented as the mean ± standard deviation, and the count data are presented as frequencies and percentages. The independent-samples *t*-test or Mann–Whitney *U* test was used for the statistical analysis of measurement data, depending on the variances to be equal or unequal. The chi-square test was used for the statistical analysis of count data.

## Results

### Basic Information of Subjects

**FIGURE 1:**
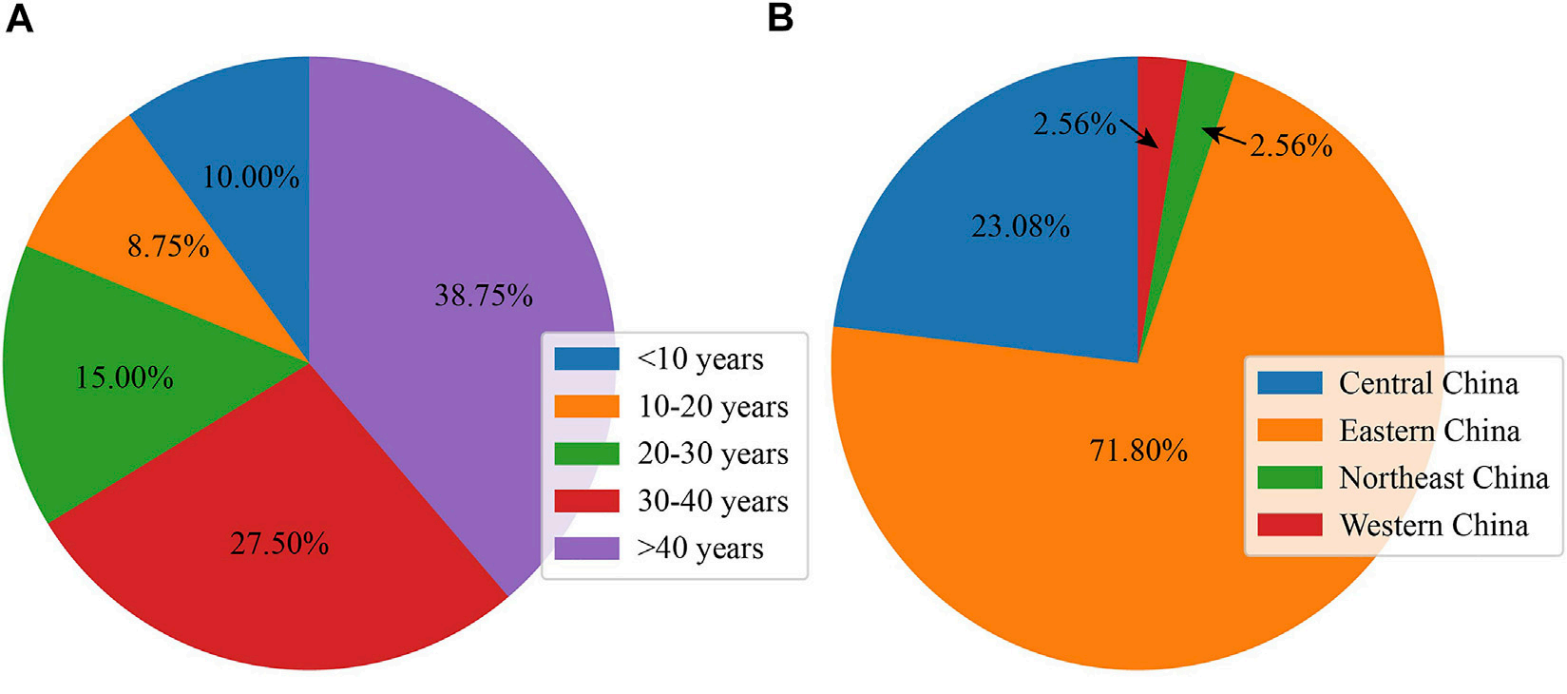
Basic and clinical information of subjects with *RPGR*-RD. **(A)** Age distribution in the Chinese cohort. **(B)** Geographic distribution in the Chinese cohort.

A total of 80 subjects (41 men and 39 women) from 39 unrelated families finally participated in this study; this cohort included 26 family cases (26 probands and their available family members) and 13 sporadic cases. As presented in Figure 1A, the age of the participants ranged from 5 to 82 years (36.35 ± 17.68 years old), and the largest proportion were more than 40 years old (38.75%, 31 subjects). As shown in Figure 1B, the majority of families were from eastern China (28 families, 71.79%), and the next largest group came from central China (9 families, 23.08%).

### Clinical Features of the Probands

As presented in the supplementary material (Supplementary Table S2), the clinical features of 38 probands (97.43%) were typical fundus features of retinal pigmentosa (Hamel, 2006), and these individuals were finally diagnosed with retinal pigmentosa type 3. In one proband, the area of photoreceptor atrophy sharply exceeded that of RPE atrophy, and the corresponding *RPGR* variant was identified as NM_001034853.1: c.3178_3179del. Among the 39 unrelated families, consanguineous marriage was confirmed in two families (family 25 and family 32), and 22 families had confirmed family histories. There were no known cases of male-to-male transmission. Among the 39 probands, the age of onset in 26 subjects (66.67%) was in childhood, and the age of onset in the remaining subjects ranged from 8 to 64 years (20.75 ± 15.72). As presented in Table 1, the duration of disease ranged from 1 to 58 years (22.68 ± 15.80) and was between 10 and 20 years in the largest percentage of cases (28.21%). In addition, the average BCVA values of the right and left eyes were 0.96 ± 0.77 and 1.00 ± 0.77, respectively. The BCVA was classified as low vision in 35.90% (14 probands) of the probands and legal blindness in 23.08% (9 probands). The detailed information of each family included in this study is presented in Supplementary Table S3 and the pedigree charts in 39 Chinese families with *RPGR*-RD are shown in Supplementary Figure S1.

**TABLE 1:** Clinical information of probands with *RPGR*-RD.

Index	Classification	Frequency/percentage (n/%)
Disease of duration	<10 years	9/23.08
	10–20 years	9/23.08
	20–30 years	3/7.69
	30–40 years	8/20.51
	>40 years	7/17.95
	uncertain	1/2.56
	**Total**	**39/100**
Vision	<0.5	14/35.90
	0.5–1.3	16/41.03
	>1.3	9/23.08
	**Total**	**39/100**

Abbreviation: RPGR-RD: RPGR-associated retinal dystrophy.

### Gene Variants

A total of 34 *RPGR* variants were identified, including 6 reported variants and 28 novel variants. The complete genetic analysis is presented in Supplementary Table S4. All identified *RPGR* variants are uploaded at a website (https://databases.lovd.nl/shared/genes/RPGR). A complex allele was detected in only one subject (no. 68, family 23, see Supplementary Table S3) and included NM_001034853.1: c.3122del, c.3119del, c.3112_3113insGAA and c.3109del. These *RPGR* variants are located very close to each other, and they can be combined in a single indel nomenclature designation: NM_001034853.1: c.3109_3122delins14. This is an in-frame indel, which is a type of indel generally considered likely to be benign. According to the ACMG classification, it was classified as a VUS. All men in this study were determined to be hemizygous for this allele by genetic analysis, and one woman (no. 71, family 24, see Supplementary Table S3) in this study was determined to be homozygous, and the remaining women were determined to be heterozygous. Furthermore, two *RPGR* variants, NM_001034853.1: c.2899_2902delGAAG and c.2744_2745ins24, were considered *de novo* variants in two patients (no. 56 and no. 59, see Supplementary Table S3) and were not found in either of their parents. The remaining 11 *RPGR* variants found in sporadic cases could not be identified as *de novo* variants due to the unavailability of appropriate family members.

According to the AGMC guideline for classifying sequence variants, the majority of the *RPGR* variants were classified as likely pathogenic, accounting for 70.59% of the variants (24 variants), and the remainder included pathogenic variants (seven variants, 20.59%) and VUS (three variants, 8.82%). The most frequent type of common nucleotide change identified in this study was deletions (16 variants, 45.06%), followed by substitutions (14 variants, 41.18%) and small insertions (four variants, 11.76%). Among these variants, 81.25% of the deletions (13 variants) were classified as likely pathogenic, and 64.29% of the substitutions (nine variants) were classified as likely pathogenic. The most common type of amino acid change identified in this study was frameshifts (17 variants, 50.00%), followed by nonsense (11 variants, 32.35%), InframeInsertion (three variants, 8.82%), missense (two variants, 5.88%) and SpliceDonor (one variant, 2.94%) mutations. Among these variants, 82.35% of the frameshifts (16 variants) were classified as likely pathogenic, and 72.73% of the nonsense variants (eight variants) were classified as likely pathogenic.

Frameshift or nonsense mutations are usually more deleterious than other types of protein function changes. In this study, five variants (NM_001034853.1: c.154G > A; c.2744_2745ins24; c.293A > G; c.3112_3113insGAA; c.2840_2841ins21) resulted in missense mutations or in-frame insertions. Among these, three variants were classified as VUS because of an extremely low incidence in healthy individuals (PM1), and the other two variants (NM_001034853.1: c.154G > A; c.2744_2745ins24) were classified as likely pathogenic. However, no functional or molecular dynamic simulation analyses of the two variants were conducted. The former variant (NM_001034853.1: c.154G > A) has been added to the HGMD database (Bukowy-Bieryłło et al., 2013). The following evidence is helpful for the classification of the latter variant (NM_001034853.1: c.2744_2745ins24): it showed an extremely low incidence in healthy individuals (PM1) and was not detected in the subject’s parents (PS2).

**FIGURE 2:**
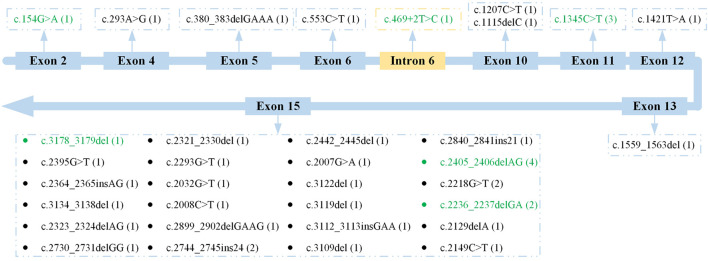
Location of all *RPGR* variants. Previously reported variants are indicated with green font. The frequency of each variant is shown in parentheses.

The locations of these *RPGR* variants are shown in Figure 2. Generally, these *RPGR* variants were distributed in 10 different subregions of *RPGR*. The genetic analysis revealed that 24 *RPGR* variants (70.59%) were distributed in exon 15; two *RPGR* variants (5.88%) were distributed in exon 10; and the remaining eight variants were distributed in exons 2, 4, 5, 6, 11, 12, and 13 and intron 6. According to analysis with Human Splicing Finder (http://www.umd.be/HSF3/), the intronic mutation mainly affected the splice donor. The *RPGR* variants distributed in exon 15 included likely pathogenic variants (17 variants, 70.83%), pathogenic variants (five variants, 20.83%), and VUS (two variants, 8.33%). In addition, deletions (13 variants, 54.17%), substitutions (seven variants, 29.17%), and insertions (four variants, 16.67%) were observed as were frameshifts (14 variants, 58.33%), nonsense mutations (seven variants, 29.17%), and in-frame insertions (three variants, 12.50%).

### High-Frequency *RPGR* Variants

Four *RPGR* variants, NM_001034853.1: c.2405_2406delAG (p. Glu802Glyfs32), c.1345C > T (p. Arg449*), c.2218G > T (p. Glu740*) and c.2236_2237delGA (p. Glu746Argfs*23), occurred at a very high frequency of 28.21% (11 families) among the 39 unrelated families a 32.50% (26 subjects) among the 80 subjects. According to the ACMG classification, these variants, including three known *RPGR* variants and one unreported *RPGR* variant (NM_001034853.1: c.2218G > T), were all classified as pathogenic. Among the 11 families with a high frequency of *RPGR* variants, 45.45% (five families) were from central China, and 45.45% (five families) were from eastern China. Only one family was from northeast China. However, among the families without a high frequency of *RPGR* variants, 82.14% were from eastern China. Finally, we classified these probands into two groups: probands with a high frequency of *RPGR* variants (11 probands) and probands with other variants (28 probands). According to the results of the chi-square test, there was a significant difference in the geographic distribution between the two groups (*p* = .000). However, neither the age of onset (*p* = .572) nor visual acuity (*p* = .274) presented a specific attentional bias in the two groups according to the results of ANOVA.

### Correlation Between Phenotype and Genotype

Twenty-seven probands were included in group A, and 12 probands were included in group B. The average BCVA values in group A and group B were 1.07 ± 0.80 and 0.72 ± 0.57, respectively. According to the results of the Mann–Whitney *U* test, there was no significant difference in the average BCVA between the two groups (*p* = .221). In the actual statistical analysis, the age of onset (from childhood) was converted to 2 years. Therefore, the average ages of onset in group A and group B were 9.63 ± 14.16 and 3.41 ± 3.48, respectively. According to the results of the Mann–Whitney *U* test, there was no significant difference in the age of onset between the two groups (*p* = .218). A total of 73.08% of the probands in group A were from eastern China, and 69.23% of the probands in group B were from eastern China. According to the results of the chi-square test, the geographic distribution (*p* = 1.000) did not present a specific attentional bias between the two groups.

## Discussion

In previous studies, a great deal of work is focused on revealing the phenotype and genotype of *RPGR*-RD (Jiang et al., 2017; Miano et al., 1999; Hu et al., 2014). Ningdong Li et al. analyze retinitis pigmentosa type 2 and *RPGR* mutations in five Han Chinese families (22). Xuyang Liu et al. identify a frameshift mutation (c.345_348delTGAA) in the *RPGR* gene of Chinese carriers (Wang et al., 2021). Enzo Di Iorio and Nobuhisa Nao-i report novel *RPGR* mutations in Italian and Japanese families, respectively (Parmeggiani et al., 2017), (Jin et al., 2006). In addition, through longitudinal follow-up, Boon, Camiel et al. investigate the clinical characteristics of patients with *RPGR*-RD in the Netherlands (Talib et al., 2018). Previous reports (Sharon et al., 2003) also reveal that individuals with *RPGR* mutations in exons 2–14 present much more severe clinical phenotypes than those with mutations in exon^ORF15^. It is postulated that *RPGR* mutations toward the 3′ end of exon ORF15 tend to be associated with cone–rod degeneration (Vervoort et al., 2000). Boon et al. also indicates that patients in the Netherlands with *RPGR-ORF15* mutations show a faster visual field decline and thinner central retina than patients with mutations in exons 1 to 14. (Talib et al., 2018).

In this study, a retrospective analysis was performed on 80 subjects with *RPG*R-RD to perform detailed genetic and clinical characterization. The analysis was based on a Chinese cohort, and the subjects came from across the country. According to the results, we drew the following conclusions. First, the greatest proportion of subjects in this study (38.75%) were more than 40 years old, and the majority of the families were from eastern China (28 families, 71.79%). This may be related to the vibrant economy of eastern China, which has increased the level of access to health care. Through further analysis, it was also found that the geographic distribution was a key factor to some extent. When we classified these probands into those with a high frequency of *RPGR* variants and those with other variants, a significant difference in the geographic distribution was observed between the two groups. However, when we classified these probands according to the distribution of variants (variants distributed in exon 15 and variants distributed in other regions), the geographic distribution between the two groups did not present a specific attentional bias. Second, we revealed that 97.43% of the identified *RPGR* variants caused XLRP disease, which was not entirely consistent with data from other regions. According to the data published in HGMD, 303 *RPGR* mutations have been identified, and 198 of these mutations are associated with XLRP. In a previous study, Xinhua Shun et al. found that 95% of *RPGR* mutations are associated with XLRP in Caucasians. However, observations made in a large cohort in the Netherlands indicate fewer retinitis pigmentosa phenotypes (70%) and more patients diagnosed with X-linked cone–rod dystrophy (23%) or cone dystrophy (7%) (Talib et al., 2018). Third, our genetic analysis identified 28 novel *RPGR* variants, which may further expand the known mutational spectrum of *RPGR*. The majority of the variants (70.59%) were classified as likely pathogenic. The most common nucleotide changes and amino acid changes identified in this study were deletions and frameshifts, respectively. Our results are based on a Chinese cohort. Although Junxin Yang et al. also reports a genotype–phenotype analysis of *RPGR* variations in a Chinese cohort (Yang et al., 2021), their results mainly reveal that most of the pathogenic variants of RPGR are truncations. Our genetic analysis reveals that these variants are distributed in 10 different subregions of *RPGR,* and 70.59% of the variants are distributed in exon 15. Our analysis did not show that subjects presented a better phenotype, better vision, or a later age of onset when the variants were located in exon 15. Overall, these data are not entirely consistent with findings in other populations. Vervoort et al. found that mutations in exon 15 account for 60% of XLRP cases in European populations (Vervoort et al., 2000) but only 22% in North American populations (Breuer et al., 2002) and 55% in Caucasians. Nobuhisa Nao-i (Jin et al., 2006) reports that two age-matched brothers with the same *RPGR* mutation presented differences in visual impairment. Thus, heterogeneity seems to be an outcome of population differences or environmental factors.

This study has certain limitations. First, the detection method is only applicable to point mutations and small mutations of no more than 20 bp, whereas neither large fragment heterozygous insertion mutations nor other special types of mutations can be detected. Sanger sequencing should be applied to validate false positive mutations. Next, the results obtained in this study are not applicable to other populations.

## Conclusion

In summary, we reveal the mutational spectrum in a Chinese cohort, which may be distinct from that in other populations. First, we identified 28 novel variants of *RPGR,* which may further expand the known mutational spectrum of *RPGR*. Among these variants, NM_001034853.1: c.2899_2902delGAAG and c.2744_2745ins24 were considered *de novo* variants. In addition, four variants, NM_001034853.1: c.2405_2406delAG, c.1345C > T, c.2218G > T and c.2236_2237delGA, occurred at a very high frequency in this Chinese cohort. Finally, 70.59% of the *RPGR* variants were distributed in exon 15. These data may provide a new reference for the genetic diagnosis of *RPGR* variants.

## Data Availability

The original contributions presented in the study are included in the article/Supplementary Material, further inquiries can be directed to the corresponding author.
