# Supplementary material for: Mutation Analysis of the RPGR Gene in a Chinese Cohort
Source: Front Genet. 2022 Mar 31;13:850122. doi: 10.3389/fgene.2022.850122 (PMC9008860; doi:10.3389/fgene.2022.850122)
Supplement: Supplementary file 1 [file DataSheet2.PDF]

Table S2. Basic information of the probands in this study

No.	Gender	Age	Family history	Geographic distribution	Consanguineous marriage	Age at symptom onset	Disease duration	BCVA（OD）	BCVA（OS）	Nucleotide change	Clinical diagnosis	Variants classification
1	Male	46	No	Eastern China	No	From childhood	46	2.3	2.3	c.1559_1563del	Retinal pigmentosa type 3	Likely pathogenic
2	Female	9	Yes	Central China	No	8	1	0.4	0.4	c.1421T>A	Retinal pigmentosa type 3	Likely pathogenic
3	Male	33	Yes	Eastern China	No	From childhood	33	0.7	0.7	c.2149C>T	Retinal pigmentosa type 3	Likely pathogenic
4	Male	28	Yes	Eastern China	No	13	15	0.6	0.9	c.293A>G	Retinal pigmentosa type 3	VUS
5	Male	38	No	Eastern China	No	27	11	1.1	1	c.3178_3179del	Macular degeneration	Likely pathogenic
6	Male	25	No	Eastern China	No	12	13	0.2	0.2	c.2395G>T	Retinal pigmentosa type 3	Pathogenic
7	Male	14	No	Central China	No	From childhood	14	0.8	0.7	c.2364_2365insAG	Retinal pigmentosa type 3	Likely pathogenic
8	Male	11	Yes	Eastern China	No	6	5	2.3	2.3	c.3134_3138del	Retinal pigmentosa type 3	Likely pathogenic
9	Female	28	Yes	Eastern China	No	uncertain	uncertain	0	0	c.2323_2324delAG	Retinal pigmentosa type 3	Likely pathogenic
10	Male	13	No	Eastern China	No	From childhood	13	0.2	0.2	c.1115delC	Retinal pigmentosa type 3	Likely pathogenic
11	Male	35	Yes	Western China	No	From childhood	35	0.9	0.8	c.154G>A	Retinal pigmentosa type 3	Likely pathogenic
12	Male	27	Yes	Eastern China	No	From childhood	27	1.5	1.3	c.2730_2731delGG	Retinal pigmentosa type 3	Pathogenic
13	Male	29	Yes	Eastern China	No	13	16	0.5	0.3	c.2321_2330del	Retinal pigmentosa type 3	Likely pathogenic
14	Female	38	No	Eastern China	No	33	5	0.7	0.6	c.2293G>T	Retinal pigmentosa type 3	Likely pathogenic
15	Male	16	No	Eastern China	No	From childhood	16	0.3	0.2	c.2032G>T	Retinal pigmentosa type 3	Likely pathogenic
16	Female	10	Yes	Eastern China	No	From childhood	10	0.3	0.3	c.2008C>T	Retinal pigmentosa type 3	Likely pathogenic
17	Female	25	No	Eastern China	No	From childhood	25	0.4	0.5	c.2899_2902delGAAG	Retinal pigmentosa type 3	Likely pathogenic
18	Male	34	No	Central China	No	From childhood	34	2.3	2.3	c.2744_2745ins24	Retinal pigmentosa type 3	Likely pathogenic
19	Male	32	No	Eastern China	No	12	10	0.5	0.8	c.2442_2445del	Retinal pigmentosa type 3	Likely pathogenic
20	Male	6	No	Eastern China	No	From childhood	6	0.6	0.5	c.1207C>T	Retinal pigmentosa type 3	Likely pathogenic
21	Male	23	No	Eastern China	No	From childhood	23	0.4	0.3	c.380_383delGAAA	Retinal pigmentosa type 3	Likely pathogenic
22	Male	58	No	Eastern China	No	From childhood	58	2	2	c.2007G>A	Retinal pigmentosa type 3	Likely pathogenic
23	Female	51	No	Eastern China	No	20	31	0.6	0.7	c.3109_3122delins14	Retinal pigmentosa type 3	Likely pathogenic
24	Female	66	No	Eastern China	No	64	2	0.3	0.4	c.2840_2841ins21	Retinal pigmentosa type 3	VUS
25	Male	40	Yes	Eastern China	Yes	From childhood	40	2.3	2	c.2744_2745ins24	Retinal pigmentosa type 3	Likely pathogenic
26	Male	41	Yes	Central China	No	From childhood	41	0.9	1	c.469+2T>C	Retinal pigmentosa type 3	VUS
27	Male	13	Yes	Central China	No	From childhood	13	0.3	1.3	c.1345C>T	Retinal pigmentosa type 3	Likely pathogenic
28	Male	7	Yes	Eastern China	No	From childhood	7	0.5	0.4	c.1345C>T	Retinal pigmentosa type 3	Pathogenic
29	Male	12	Yes	Eastern China	No	From childhood	12	0.6	0.5	c.1345C>T	Retinal pigmentosa type 3	Pathogenic
30	Female	29	Yes	Central China	No	22	7	0.4	0.4	c.2405_2406delAG	Retinal pigmentosa type 3	Pathogenic
31	Male	32	Yes	Eastern China	No	From childhood	32	2.3	2.3	c.2405_2406delAG	Retinal pigmentosa type 3	Pathogenic
32	Male	42	Yes	Eastern China	Yes	From childhood	42	1.3	2.3	c.2405_2406delAG	Retinal pigmentosa type 3	Pathogenic
33	Male	49	Yes	Central China	No	From childhood	49	1.5	1.4	c.2405_2406delAG	Retinal pigmentosa type 3	Pathogenic
34	Male	64	No	Northeast China	No	19	45	2.6	2.6	c.2218G>T	Retinal pigmentosa type 3	Pathogenic
35	Male	33	Yes	Central China	No	From childhood	33	1	0.8	c.2218G>T	Retinal pigmentosa type 3	Pathogenic
36	Male	6	Yes	Central China	No	From childhood	6	0.7	0.6	c.2236_2237delGA	Retinal pigmentosa type 3	Pathogenic
37	Male	45	Yes	Eastern China	No	From childhood	45	2.3	2.3	c.2236_2237delGA	Retinal pigmentosa type 3	Pathogenic
38	Male	6	No	Eastern China	No	From childhood	6	0.4	0.4	c.553C>T	Retinal pigmentosa type 3	Likely pathogenic
39	Male	35	Yes	Eastern China	No	From childhood	35	0.5	0.9	c.2129delA	Retinal pigmentosa type 3	Likely pathogenic
