# Supplementary material for: Mutation Analysis of the RPGR Gene in a Chinese Cohort
Source: Front Genet. 2022 Mar 31;13:850122. doi: 10.3389/fgene.2022.850122 (PMC9008860; doi:10.3389/fgene.2022.850122)
Supplement: Supplementary file 2 [file DataSheet4.PDF]

Table S4. ***RPGR*** **variants identified in this study and their in silico functional assessment**

NO.	Gene	Transcript	Nucleotide change	Amino acid change	Exon/Intron ID	Chr:Location	dbSNP ID	1000 Genomes	dbSNP	ESP6500	ExAC	GnomAD	FunctionalChange	SIFT	Polyphen2	MutationTaster	Previous Reports
1	RPGR	NM_001034853.1	c.1559_1563del	p.Val520Glufs19	EX13	chrX:38290967:TCTGAA>T	-	0	0	0	0	0	Frameshift	-	-	-	-
2	RPGR	NM_001034853.1	c.1421T>A	p.Leu474*	EX12	chrX:38291478:A>T	-	0	0	0	0	0	Nonsense	-	-	Disease_causing	-
3	RPGR	NM_001034853.1	c.2149C>T	p.Gln717*	EX15	chrX:38286850:G>A	-	0	0	0	0	0	Nonsense	-	-	Disease_causing	-
4	RPGR	NM_001034853.1	c.293A>G	p.His98Arg	EX4	chrX:38321044:T>C	-	0	0	0	0	0	Missense	Deleterious	-	Disease_causing	-
5	RPGR	NM_001034853.1	c.3178_3179del	p.Glu1060Argfs18	EX15	chrX:38285819:TTC>T	rs771214648	0	0	0.0028	0.00001647	0.0000113435	Frameshift	-	-	-	Lee H et.al 2014
6	RPGR	NM_001034853.1	c.2395G>T	p.Glu799*	EX15	chrX:38286604:C>A	-	0	0	0	0	0	Nonsense	-	-	Disease_causing	-
7	RPGR	NM_001034853.1	c.2364_2365insAG	p.Glu789Argfs27	EX15	chrX:38286634:C>CCT	-	0	0	0	0	0	Frameshift	-	-	-	-
8	RPGR	NM_001034853.1	c.3134_3138del	p.Glu1045Glyfs32	EX15	chrX:38285860:CCCTTT>C	-	0	0	0	0	0	Frameshift	-	-	-	-
9	RPGR	NM_001034853.1	c.2323_2324delAG	p.Arg775Glufs59	EX15	chrX:38286674:CCT>C	-	0	0	0	0	0	Frameshift	-	-	-	-
10	RPGR	NM_001034853.1	c.1115delC	p.Ala372Glufs9	EX10	chrX:38299085:TG>T	-	0	0	0	0	0	Frameshift	-	-	-	-
11	RPGR	NM_001034853.1	c.154G>A	p.Gly52Arg	EX2	chrX:38323399:C>T	-	0	0	0	0	0	Missense	Tolerated	-	Disease_causing	Bukowy-Bieryllo Z et.al 2013
12	RPGR	NM_001034853.1	c.2730_2731delGG	p.Glu911Glyfs*167	EX15	chrX:38286267:TCC>T	-	0	0	0	0	0	Frameshift	-	-	-	-
13	RPGR	NM_001034853.1	c.2321_2330del	p.Glu774Glyfs38	EX15	chrX:38286668:CTTTTTCCTCT>	-	0	0	0	0	0	Frameshift	-	-	-	-
14	RPGR	NM_001034853.1	c.2293G>T	p.Glu765*	EX15	chrX:38286706:C>A	-	0	0	0	0	0	Nonsense	-	-	Disease_causing	-
15	RPGR	NM_001034853.1	c.2032G>T	p.Glu678*	EX15	chrX:38286967:C>A	-	0	0	0	0	0	Nonsense	-	-	Disease_causing	-
16	RPGR	NM_001034853.1	c.2008C>T	p.Gln670*	EX15	chrX:38286991:G>A	-	0	0	0	0	0	Nonsense	-	-	Disease_causing	-
17	RPGR	NM_001034853.1	c.2899_2902delGAAG	p.Glu967Argfs121	EX15	chrX:38286096	rs753842840	0	0	0	0.00008796	0.0000308814	Frameshift	-	-	-	-
18	RPGR	NM_001034853.1	c.2442_2445del	p.Gly817Lysfs2	EX15	chrX:38286553:CCTCT>C	-	0	0	0	0	0	Frameshift	-	-	-	-
19	RPGR	NM_001034853.1	c.1207C>T	p.Gln403Ter	EX10	chrX:38298994:G>A	-	0	0	0	0	0	Nonsense	-	-	Disease_causing	-
20	RPGR	NM_001034853.1	c.380_383delGAAA	p.Arg127Thrfs5	EX5	chrX:38318914:GTTTC>G	-	0	0	0	0	0	Frameshift	-	-	-	-
21	RPGR	NM_001034853.1	c.2007G>A	p.Trp669Ter	EX15	chrX:38286992:C>T	-	0	0	0	0	0	Nonsense	-	-	Disease_causing	-
22	RPGR	NM_001034853.1	c.3122del	p.Glu1041Glyfs48	EX15	chrX:38285876:CT>C	rs761610612	0	0	0	0	0	Frameshift	-	-	-	-
23	RPGR	NM_001034853.1	c.3119del	p.Glu1040Glyfs49	EX15	chrX:38285879:CT>C	rs767219869	0	0	0	0.00002503	0	Frameshift	-	-	-	-
24	RPGR	NM_001034853.1	c.3112_3113insGAA	p.1038_1039insGly	EX15	chrX:38285886:T>TTTC	rs766460691	0	0	0	0.00001663	0.0000426587	InframeInsertion	-	-	-	-
25	RPGR	NM_001034853.1	c.3109del	p.Glu1037Argfs52	EX15	chrX:38285889:TC>T	rs752785210	0	0	0	0	0.000043406	Frameshift	-	-	-	-
26	RPGR	NM_001034853.1	c.2840_2841ins21	p.Glu947_Gly948insProLe	EX15	chrX:38286158:C>CTCCCCTTCT	rs764268405	0	0	0	0.008457	0	InframeInsertion	-	-	-	-
27	RPGR	NM_001034853.1	c.2744_2745ins24	p.Glu915_Glu916insProLe	EX15	chrX:38286254:C>CTCCTCTTCT	-	0	0	0	0	0.03312	InframeInsertion	-	-	-	-
28	RPGR	NM_001034853.1	c.469+2T>C	-	Intron6	chrX:38318827:A>G	-	0	0	0	0	0	Intron+SpliceDonor	-	-	Disease_causing	Branham et.al 2012
29	RPGR	NM_001034853.1	c.1345C>T	p.Arg449*	EX11	chrX:38297353:G>A	-	0	0	0	0	0	Nonsense	-	-	Disease_causing	Hemangi Patil et.al 2012
30	RPGR	NM_001034853.1	c.2405_2406delAG	p.Glu802Glyfs32	EX15	chrX:38286592:CCT>C	rs398122960	0	0	0	0	0	Frameshift	-	-	-	Xu Y et.al 2014
31	RPGR	NM_001034853.1	c.2218G>T	p.Glu740*	EX15	chrX:38286781:C>A	rs983693027	0	0	0	0	0	Nonsense	-	-	Disease_causing	-
32	RPGR	NM_001034853.1	c.2236_2237delGA	p.Glu746Argfs*23	EX15	chrX:38286761:TTC>T	-	0	0	0	0	0	Frameshift	-	-	-	Meindl A et.al 2000
33	RPGR	NM_001034853.1	c.553C>T	p.Gln185*	EX6	chrX:38317382:G>A	-	0	0	0	0	0	Nonsense	-	-	Disease_causing	-
34	RPGR	NM_001034853.1	c.2129delA	p.Gln710Argfs*105	EX15	chrX:38286869:CT>C	-	0	0	0	0	0	Frameshift	-	-	-	-
