# Supplementary material for: Mutation Analysis of the RPGR Gene in a Chinese Cohort
Source: Front Genet. 2022 Mar 31;13:850122. doi: 10.3389/fgene.2022.850122 (PMC9008860; doi:10.3389/fgene.2022.850122)
Supplement: Supplementary file 4 [file DataSheet3.PDF]

Table S3. Information of each family in this study

Family NO.	Subject NO.	Gender	Age	Clinical diagnosis	Nucleotide change	Exon/Intron ID	Zygosity
NO. 1	1	Male	46	RP	c.1559_1563del	EX13	Hemi
	2	Female	24	NC	c.1559_1563del	EX13	Het
NO. 2	3	Female	9	RP	c.1421T>A	EX12	Het
	4	Female	7	RP	c.1421T>A	EX12	Het
	5	Female	31	RP	c.1421T>A	EX12	Het
	6	Male	57	RP	c.1421T>A	EX12	Hemi
	7	Male	28	RP	c.1421T>A	EX12	Hemi
	8	Female	29	RP	c.1421T>A	EX12	Het
	9	Male	31	NC	-		
NO. 3	10	Male	33	RP	c.2149C>T	EX15	Hemi
	11	Female	60	RP	c.2149C>T	EX15	Het
NO. 4	12	Male	28	RP	c.293A>G	EX4	Hemi
	13	Female	54	RP	c.293A>G	EX4	Het
	14	Male	55	NC	-		
NO. 5	15	Male	38	ST	c.3178_3179del	EX15	Hemi
	16	Female	61	NC	c.3178_3179del	EX15	Het
	17	Male	68	NC	-		
NO. 6	18	Male	25	RP	c.2395G>T	EX15	Hemi
	19	Female	47	NC	c.2395G>T	EX15	Het
	20	Male	48	NC	-		
NO. 7	21	Male	14	RP	c.2364_2365insAG	EX15	Hemi
	22	Female	35	NC	c.2364_2365insAG	EX15	Het
	23	Male	35	NC	-		
NO. 8	24	Male	11	RP	c.3134_3138del	EX15	Hemi
	25	Female	36	NC	c.3134_3138del	EX15	Het
	26	Female	61	NC	c.3134_3138del	EX15	Het
	27	Male	39	NC	-		
NO. 9	28	Female	28	RP	c.2323_2324delAG	EX15	Het
	29	Female	82	RP	c.2323_2324delAG	EX15	Het
	30	Female	60	NC	c.2323_2324delAG	EX15	Het
	31	Male	34	RP	c.2323_2324delAG	EX15	Hemi
NO. 10	32	Male	13	RP	c.1115delC	EX10	Hemi
	33	Female	33	NC	c.1115delC	EX10	Het
	34	Male	35	NC	-		
	35	Female	15	NC	-		
NO. 11	36	Male	35	RP	c.154G>A	EX2	Hemi
	37	Female	55	RP	c.154G>A	EX2	Het
	38	Male	27	NC	-		
	39	Male	60	NC	-		
NO. 12	40	Male	27	RP	c.2730_2731delGG	EX15	Hemi
	41	Female	50	RP	c.2730_2731delGG	EX15	Het
NO. 13	42	Male	29	RP	c.2321_2330del	EX15	Hemi
	43	Female	53	NC	c.2321_2330del	EX15	Het
	44	Male	50	RP	c.2321_2330del	EX15	Hemi
	45	Male	55	NC	-		
NO. 14	46	Female	38	RP	c.2293G>T	EX15	Het
	47	Male	5	NC	c.2293G>T	EX15	Hemi
	48	Male	48	NC	-		
NO. 15	49	Male	16	RP	c.2032G>T	EX15	Hemi
	50	Female	40	NC	c.2032G>T	EX15	Het
	51	Male	41	NC	-		
	52	Female	9	NC	-		
NO. 16	53	Female	10	RP	c.2008C>T	EX15	Het
	54	Male	36	RP	c.2008C>T	EX15	Hemi
	55	Female	37	NC	-		
NO. 17	56	Female	25	RP	c.2899_2902delGAAG	EX15	Het
	57	Male	50	NC	-		
	58	Female	50	NC	-		
NO. 18	59	Male	34	RP	c.2744_2745ins24	EX15	Hemi
	60	Male	60	NC	-		
	61	Female	58	NC	-		
NO. 19	62	Male	32	RP	c.2442_2445del	EX15	Hemi
NO. 20	63	Male	6	RP	c.1207C>T	EX10	Hemi
	64	Male	34	NC	-		
NO. 21	65	Male	23	RP	c.380_383delGAAA	EX5	Hemi
	66	Male	53	NC	-		
NO. 22	67	Male	58	RP	c.2007G>A	EX15	Hemi
NO. 23	68	Female	51	RP	c.3122del	EX15	Het
					c.3119del	EX15	Het
					c.3112_3113insGAA	EX15	Het
					c.3109del	EX15	Het
	69	Male	40	NC	-		
	70	Female	74	NC	-		
NO. 24	71	Female	66	RP	c.2840_2841ins21	EX15	Hom
NO. 25	72	Male	40	RP	c.2744_2745ins24	EX15	Hemi
NO. 26	73	Male	41	RP	c.469+2T>C	Intron6	Hemi
NO. 27	74	Male	13	RP	c.1345C>T	EX11	Hemi
	75	Female	45	RP	c.1345C>T	EX11	Het
NO. 28	76	Male	7	RP	c.1345C>T	EX11	Hemi
	77	Female	32	NC	c.1345C>T	EX11	Het
	78	Male	30	NC	-		
NO. 29	79	Male	12	RP	c.1345C>T	EX11	Hemi
	80	Female	33	NC	c.1345C>T	EX11	Het
	81	Male	36	NC	-		
NO. 30	82	Female	29	RP	c.2405_2406delAG	EX15	Het
	83	Male	53	RP	c.2405_2406delAG	EX15	Hemi
	84	Female	55	NC	-		
NO. 31	85	Male	32	RP	c.2405_2406delAG	EX15	Hemi
	86	Female	7	RP	c.2405_2406delAG	EX15	Het
	87	Male	56	RP	c.2405_2406delAG	EX15	Het
	88	Male	48	RP	c.2405_2406delAG	EX15	Hemi
	89	Female	54	RP	c.2405_2406delAG	EX15	Het
	90	Female	70	NC	-		
	91	Male	56	NC	-		
NO. 32	92	Male	42	RP	c.2405_2406delAG	EX15	Hemi
NO. 33	93	Male	49	RP	c.2405_2406delAG	EX15	Hemi
	94	Male	42	NC	-		
	95	Female	78	RP	c.2405_2406delAG	EX15	Het
NO. 34	96	Male	64	RP	c.2218G>T	EX15	Hemi
NO. 35	97	Male	33	RP	c.2218G>T	EX15	Hemi
	98	Female	57	RP	c.2218G>T	EX15	Het
	99	Male	34	RP	c.2218G>T	EX15	Hemi
	100	Female	64	NC	c.2218G>T	EX15	Het
	101	Male	66	NC	-		
NO. 36	102	Male	6	RP	c.2236_2237delGA	EX15	Hemi
	103	Female	28	RP	c.2236_2237delGA	EX15	Het
	104	Female	31	RP	c.2236_2237delGA	EX15	Het
	105	Female	56	RP	c.2236_2237delGA	EX15	Het
	106	Male	33	NC	-		
NO. 37	107	Male	45	RP	c.2236_2237delGA	EX15	Hemi
	108	Male	73	NC	-		
NO. 38	109	Male	6	RP	c.553C>T	EX6	Hemi
	110	Female	31	NC	c.553C>T	EX6	Het
	111	Male	31	NC	-		
NO. 39	112	Male	35	RP	c.2129delA	EX15	Hemi
	113	Female	60	NC	c.2129delA	EX15	Het
	114	Male	62	NC	-		
