# Supplementary material for: Mutation Analysis of the RPGR Gene in a Chinese Cohort
Source: Front Genet. 2022 Mar 31;13:850122. doi: 10.3389/fgene.2022.850122 (PMC9008860; doi:10.3389/fgene.2022.850122)
Supplement: Supplementary file 5 [file DataSheet1.PDF]

Table S1. List of genes in the capture panel.

792 genes involved in common inherited eye diseases in the panel
SRY, ABCA4, ABCB6, ABCC2, ABCC6, ABHD12, ACBD5, ACO2, ACTA1, ACTB, ACTG1, ADAM9, ADAMTS10, ADAMTS17, ADAMTS18, ADAMTSL4, ADAR, ADGRA3, ADGRV1, ADIPOR1, AGBL5, AGK, AHI1, AIPL1, ALMS1, ANAPC1, ANO5, AP3B1, APTX, ARL13B, ARL2BP, ARL3, ARL6, ARMS2, ASRGL1, ATF6, ATP13A2, ATP2C1, ATXN10, ATXN7, B3GALNT2, B3GLCT, B4GAT1, B9D1, BAP1, BBIP1, BBS1, BBS10, BBS12, BBS2, BBS4, BBS5, BBS7, BBS9, BCOR, BEST1, BFSP1, BFSP2, BLOC1S3, BLOC1S6, BMP4, BRAF, C10orf11, C10orf2, C12orf65, C1QTNF5, C2, C21orf2, C2orf71, C3, C5orf42, C8orf37, C9, CA4, CABP4, CACNA1F, CACNA2D4, CAPN3, CAPN5, CAV3, CBS, CC2D2A, CDH23, CDH3, CDHR1, CEP164, CEP250, CEP290, CEP41, CERKL, CFB, CFH, CFI, CFL2, CHD7, CHM, CHMP4B, CHN1, CHST6, CIB2, CISD2, CLN3, CLN5, CLN6, CLN8, CLRN1, CLUAP1, CNBP, CNGA1, CNGA3, CNGB1, CNGB3, CNNM4, COL11A1, COL11A2, COL18A1, COL2A1, COL4A1, COL6A1, COL6A2, COL6A3, COL9A1, COL9A2, CRB1, CRX, CRYAA, CRYAB, CRYBA1, CRYBA2, CRYBA4, CRYBB1, CRYBB2, CRYBB3, CRYGB, CRYGC, CRYGD, CRYGS, CSPP1, CST3, CTC1, CTDP1, CTNNA1, CTSD, CTSF, CX3CR1, CYP1B1, CYP4V2, DCN, DHDDS, DHX38, DMD, DMPK, DNA2, DNAJC5, DRAM2, DRD5, DTHD1, DTNBP1, DUX4, DYSF, EDN3, EDNRB, EFEMP1, ELOVL4, EMC1, EMD, EPHA2, ERCC1, ERCC2, ERCC6, ERCC8, EXOSC2, EYS, FAM126A, FAM161A, FBLN5, FBN1, FGF10, FGFR1, FGFR2, FGFR3, FHL1, FKRP, FKTN, FLVCR1, FOXC1, FOXE3, FOXL2, FRAS1, FREM1, FREM2, FRMD7, FSCN2, FTL, FYCO1, FZD4, GABRB1, GALC, GCNT2, DF3, GDF6, GFER, GJA1, GJA3, GJA8, GJB2, GMPPB, GNAT1, GNAT2, GNB3, GNPTG, GP1BA, GPR143, GPR179, GRIP1, GRK1, GRM6, GRN, GUCA1A, GUCA1B, GUCY2D, HARS, HCCS, HESX1, HFE, HGSNAT, HK1, HMCN1, HMGB3, HMX1, HPS1, HPS3, HPS4, HPS5, HPS6, HSF4, HTRA1, IDH3B, IDUA, IFT140, IFT172, IFT27, IGBP1, IKBKG, IMPDH1, IMPG1, IMPG2, INPP5E, INVS, IQCB1, ISPD, ITGA2B, ITGA7, ITGB3, ITM2B, JAG1, JAM3, KCNJ10, KCNJ13, KCNV2, KCTD7, KERA, KIAA1549, KIF11, KIF21A, KIF7, KIT, KIZ, KLHL7, KMT2D, KRAS, KRT12, KRT3, LAMA1, LAMA2, LAMB2, LCA5, MTND1, LIM2, LMNA, LOXL1, LRAT, LRIT3, LRP5, LTBP2, LYST, LZTFL1, MAB21L2, MAF, MAK, MAP2K1, MAPKAPK3, MAPT, MC1R, MCOLN1, MERTK, MFN2, MFRP, MFSD8, MIP, MIR204, MITF, MKKS, MKS1, MT-ATP6, MTM1, MT-TH, MT-TL1, MTTP, MT-TP, MTTS2, MVK, MYH7, MYO7A, MYOC, MYOT, NBAS, NDP, NEB, NEK2, NEUROD1, NHS, NMNAT1, NOD2, NPHP1, NPHP3, NPHP4, NR2E3, NR2F1, NRAS, NRL, NTF4, NYX, OAT, OCA2, OCRL, OFD1, OPA1, OPA3, OPN1LW, OPN1MW, OPN1SW, OPTN, OR2W3, OTX2, PABPN1, PANK2, PAX2, PAX3, PAX6, PCDH15, PCYT1A, PDE6A, PDE6B, PDE6C, PDE6G, PDE6H, PDZD7, PEX1, PEX2, PEX7, PGK1, PHOX2A, PHYH, PIGL, PIKFYVE, PITPNM3, PITX2, PITX3, PLA2G5, PLEC, PLG, PLK4, PNPLA6, POC1B, POLG, POLG2, POMGNT1, POMGNT2, POMK, POMT1, POMT2, PPT1, PRCD, PRDM13, PRDM5, PROM1, PRPF3, PRPF31, PRPF4, PRPF6, PRPF8, PRPH2, PRPS1, PRSS56, PTPN11, RAB18, RAB28, RAB3GAP1, RAB3GAP2, RAF1,

RARB, RAX, RAX2, RB1, RBP3, RBP4, RCBTB1, RD3, RDH11, RDH12, RDH5, RGR, RGS9, RGS9BP, RHO, RIMS1, RLBP1, ROM1, RP1, RP1L1, RP2, RP9, RPE65, RPGR, RPGRIP1, RPGRIP1L, RRM2B, RS1, RTN4IP1, RYR1, SAG, SALL1, SDCCAG8, SEMA4A, SEPN1, SETX, SGCA, SGCB, SGCD, SGCG, SHH, SHOX, SIL1, SIX6, SLC16A12, SLC24A1, SLC24A5, SLC25A4, SLC25A46, SLC26A4, SLC45A2, SLC4A11, SLC4A4, SLC7A14, SMOC1, SNAI2, SNRNP200, SOS1, SOX10, SOX2, SPATA7, SPP2, STRA6, SYNE1, SYNE2, TACSTD2, TBC1D20, TCAP, TCOF1, TCTN1, TCTN2, TCTN3, TDRD7, TEAD1, TFAP2A, TGFBI, TIMM8A, TIMP3, TINF2, TLR4, TMEM126A, TMEM138, TMEM216, TMEM231, TMEM237, TMEM5, TMEM67, TNNT1, TOPORS, TP63, TPM2, TPM3, TPP1, TREX1, TRIM32, TRIM37, TRNT1, TRPM1, TSPAN12, TTC21B, TTC8, TTLL5, TTN, TTPA, TTR, TUB, TUBA8, TUBB3, TUBGCP4, TUBGCP6, TULP1, TYR, TYRP1, UBIAD1, UNC119, USH1C, USH1G, USH2A, VAX1, VCAN, VIM, VSX1, VSX2, WDPCP, WDR19, WDR36, WFS1, ZNF408, ZNF423, ZNF469, ZNF513, ABCA3, ABHD5, ACD, ACVRL1, AFG3L2, AGPS, AGRN, AGXT, ALDH18A1, ALDH1A3, , ALDH3A2, ANO10, ANTXR1, AP4M1, AP5Z1, APC, ARSB, ARSE, ASAH1, ASB10, ASPM, ATL1, ATM, ATP6V0A2, ATP7A, ATP7B, AUH, B4GALNT1, BLM, C19orf12, CCM2, CDK5RAP2, CENPJ, CEP135, CEP152, CHAT, CHMP1A, CHRNG, CLCN7, COASY, COL3A1, COL4A4, COL4A5, COLEC11, COLQ, COX10, COX15, COX7B, CREBBP, CTSA, CUBN, CYLD, CYP27A1, CYP2U1, CYP7B1, DAG1, DBH, DDHD2, DDX59, DHCR7, DHODH, DNAJC19, DNM2, DOK7, EBP, EDARADD, EEF2, EFEMP2, EP300, EPG5, ERCC3, ERLIN2, ESCO2, EXOSC3, FA2H, FAH, FLNA, FMR1, FUCA1, GALE, GALK1, GALNS, GALT, GBA, GBA2, GCM2, GDF2, GFAP, GLB1, GM2A, GNAS, GNPAT, GNS, GRHPR, GUSB, HDAC8, HEXA, HGD, HOGA1, HPD, HSPD1, HSPG2, HYAL1, IDS, IKBKAP, IRF6, ITPR1, KCNC3, KCND3, KCNH2, KCNJ2, KCNQ1, KDM6A, KIAA0196, KIF1A, KIF1BP, KIF5A, LAMA3, LAMB3, LAMC2, LARGE1, LCAT, LMX1B, LRPAP1, LTBP4, MAN2B1, MANBA, MARS2, MCPH1, MID1, MLPH, MMACHC, MRE11A, MSH2, MTPAP, NAGLU, NEU1, NF2, NFIX, NHP2, NIPA1, NIPBL, NOP10, NOTCH2, NPC1, NPC2, NSD1, OSTM1, PDK3, PEX10, PEX11B, PEX12, PEX13, PEX14, PEX16, PEX26, PEX5, PEX6, PHF6, PIK3R1, PKP1, PLA2G6, PLOD1, POLR1C, PORCN, PRIMPOL, PRKCG, PRX, PTCH1, PTCH2, PTH, PYCR1, RAB27A, RAD21, RARS2, RECQL4, REEP1, RNASEH1, ROBO3, RTN2, SALL4, SCN4A, SEPSECS, SF3B4, SLC33A1, SLITRK6, SMC1A, SMPD1, SNX10, SOD2, SPG11, SPG7, SPINK5, SPTBN2, STIL, STK11, STS, SURF1, SYT14, TACO1, TAT, TBX1, TCIRG1, TERT, TFAP2B, TGFBR1, TGM6, TLR1, TLR2, TNFRSF11A, TNFSF11, TNXB, TRPV4, TSC1, TSC2, TSEN2, TSEN34, TSEN54, TTBK2, TWIST1, UCHL1, UROD, VHL, VRK1, WHRN, WRAP53, WRN, WWOX, XPA, XPC, ZFYVE26, ZFYVE27, ZNF335, ZNF644, ACTA2, ATP1A3, CAV1, CHRDL1, CNTN1, COL8A2, FBN2, IARS2, LONP1, MPZ, MSMO1, MSTN, MYH11, MYLK, NAA10, P3H2, PRKG1, PXDN, SEMA3E, SLC2A10, SMAD3, TCF4, TGFB2, TGFB3, TGFBR2, ZEB1
